# Regulatory Networks Controlling Nitrogen Sensing and Uptake in *Candida albicans*

**DOI:** 10.1371/journal.pone.0092734

**Published:** 2014-03-20

**Authors:** Shruthi Ramachandra, Jörg Linde, Matthias Brock, Reinhard Guthke, Bernhard Hube, Sascha Brunke

**Affiliations:** 1 Department of Microbial Biochemistry and Physiology, Leibniz Institute for Natural Product Research and Infection Biology – Hans Knoell Institute, Jena, Germany; 2 Department of Systems Biology & Bioinformatics, Leibniz Institute for Natural Product Research and Infection Biology – Hans Knoell Institute, Jena, Germany; 3 Department of Microbial Pathogenicity Mechanisms, Leibniz Institute for Natural Product Research and Infection Biology – Hans Knoell Institute, Jena, Germany; 4 Center for Sepsis Control and Care (CSCC), Jena University Hospital, Jena, Germany; 5 Friedrich Schiller University, Jena, Germany; Louisiana State University, United States of America

## Abstract

Nitrogen is one of the key nutrients for microbial growth. During infection, pathogenic fungi like *C. albicans* need to acquire nitrogen from a broad range of different and changing sources inside the host. Detecting the available nitrogen sources and adjusting the expression of genes for their uptake and degradation is therefore crucial for survival and growth as well as for establishing an infection. Here, we analyzed the transcriptional response of *C. albicans* to nitrogen starvation and feeding with the infection-relevant nitrogen sources arginine and bovine serum albumin (BSA), representing amino acids and proteins, respectively. The response to nitrogen starvation was marked by an immediate repression of protein synthesis and an up-regulation of general amino acid permeases, as well as an up-regulation of autophagal processes in its later stages. Feeding with arginine led to a fast reduction in expression of general permeases for amino acids and to resumption of protein synthesis. The response to BSA feeding was generally slower, and was additionally characterized by an up-regulation of oligopeptide transporter genes. From time-series data, we inferred network interaction models for genes relevant in nitrogen detection and uptake. Each individual network was found to be largely specific for the experimental condition (starvation or feeding with arginine or BSA). In addition, we detected several novel connections between regulator and effector genes, with putative roles in nitrogen uptake. We conclude that *C. albicans* adopts a particular nitrogen response network, defined by sets of specific gene-gene connections for each environmental condition. All together, they form a grid of possible gene regulatory networks, increasing the transcriptional flexibility of *C. albicans*.

## Introduction

The ability to use a broad range of nutrients is essential for most living beings. To this end, organisms must be able to quickly adapt to the changing availability of nutrients by regulating their uptake and metabolic pathways accordingly. This is especially true for opportunistic pathogens during their transition from a commensal or environmental life style to pathogenic interactions within a susceptible host [1], [2]. In fact, nutrient acquisition can be considered one of the most important pathogenicity factors of pathogens, including the fungus *Candida albicans* which causes superficial to deep-seated infections in humans [3]. As a facultative pathogen, *C. albicans* must be able to adapt to the dynamic nutrient niches presented by the host and the cohabitating microflora of mucosal surfaces in its commensal stage. However, in order to establish itself successfully as a pathogen, *C. albicans* must be able to utilize distinct nutrients from the host itself [1].

Overall, carbon and nitrogen compounds are the main sources for biosynthetic processes and energy. While other elements like iron [4], [5] or zinc [6] are required in very small amounts [7] and are therefore considered micronutrients, these macronutrients must be obtained from the host in comparatively large quantities. For example, in order to survive and grow within the host, *C. albicans* is able to utilize various fermentable and non-fermentable carbon sources [8], [9]. Even in the presence of glucose, other, non-fermentable sugars, fatty acids, or amino acids can serve as sources of carbon [1], [10].

In addition, fungi can also use a surprisingly diverse range of nitrogen sources. Ammonia, glutamine, asparagine, and glutamate are preferentially used [11]. However, when these primary nitrogen sources are limited or not available, fungi can switch to the use of less preferred sources like other amino acids, polyamines, or start to hydrolyze proteins [11]–[13]. Thus, during infection *C. albicans* expresses various extracellular hydrolytic enzymes, such as the secreted aspartic proteases (Saps) [14]. Liberated oligopeptides and amino acids are then taken up by dedicated oligopeptide transporters (Opt1-8) [15] and a family of 22 predicted amino acid permeases [16], [17], respectively. When expressed (at the right time) in host tissue, these factors enable *C. albicans* to use a broad range of nitrogen sources over the course of an infection.

Expression of these genes is dependent on the ability to sense the available nitrogen sources. Similar to *S. cerevisiae* [18], this occurs mainly via the SPS sensor mechanism, comprising the amino acid receptor Ssy1, the scaffold protein Ptr3, and the signaling endopeptidase Ssy5. The proteolytic processing of the initially inert transcription factors, Stp1 and Stp2, then leads to their nuclear translocation and activation [12], [19]. This system allows the fungus to detect the presence of nitrogen sources, and regulate a plethora of specific target genes. In addition, *C. albicans* likely has more regulators available to modify its gene expression profile according to the available nitrogen sources. Understanding these molecular networks will provide us with a better picture of the nutrient uptake processes, the interplay between the genes involved in the metabolism of nitrogen sources, and finally, the possible role of these genes in pathogenesis.

A highly suitable tool to understand these regulatory networks is network inference [20], [21]. This systems biology approach aims at predicting regulatory interactions based on -omics data (most often gene expression data). To this end, current approaches integrate prior knowledge – interactions known from literature or predicted by consulting other data sources – with transcriptional data to improve the reliability of the predicted interactions. Even though there are a number of different approaches [20], network inference can be broadly divided into three major steps: 1) identifying effector genes and their potential regulators, 2) collecting known interactions (“prior knowledge”) between and among these two groups, based on information from different data sources, like literature or homology data, and 3) mathematical modeling integrating -omics data and prior knowledge. This approach results in the prediction of novel interactions, which can then be tested in the ‘wet’ laboratory – for example by classical gene deletion and phenotyping strategies. In our previous work, we have successfully predicted and validated regulatory interactions of human fungal pathogens in response to iron limitation and in host-fungus interactions [22]–[24].

In this study, we inferred different transcriptional networks of *C. albicans* in the absence or presence of different nitrogen sources, broadly simulating typical situations in the host. We obtained the transcriptional profiles of the fungus during nitrogen starvation and after feeding pre-starved cells with proteins or amino acids. We compared these inferred regulatory interaction networks and identified core regulatory mechanisms and the overall network flexibility.

## Materials and Methods

### Strains and culture conditions

Wild-type *C. albicans* (SC5314) were routinely grown in YPD complex medium (1% yeast extract, 2% peptone, 2% glucose) at 30°C with shaking at 200 rpm. For feeding experiments, minimal medium (4 g KH_2_PO_4_, 3.2 g NaH_2_PO_4_, 5 g (NH_4_)_2_SO_4_, 0.7 g MgSO_4_·7H_2_O per litre, pH 6.5 [25], [26]) supplemented with 0.03% trace mineral solution (composition per 100 ml: 0.5 g CuSO_4_·5H_2_O, 0.5 g ZnSO_4_·7H_2_O, 0.8 g MnCl_2_·4H_2_O, 0.5 g FeSO_4_, in 0.1 M HCl), 0.12% vitamin solution (composition per 100 ml: 2 mg biotin, 20 mg thiamine-HCl, 20 mg pyridoxine-HCl in 20% ethanol) and 2% glucose was used. The medium was either used directly without nitrogen source for starvation, or with 0.1% w/v bovine serum albumin (BSA), 0.1% w/v casamino acids (CAA), 0.5% ammonium sulphate, 10 mM urea, or 10 mM amino acid as nitrogen source.

### Growth and hyphae formation tests

Growth of *C. albicans* was followed by measuring the OD_600_ of 200 μl culture in 96-well plates in a Fluostar Omega microplate reader (BMG LabTech), with intermittent shaking. Hyphae formation was measured by visually inspecting 200 fungal cells for each time-point under the microscope for the presence of hyphae or germ tubes.

### Transcriptional profiling

For the transcription profiles, *C. albicans* were grown over night at 30°C in YPD, transferred to fresh YPD at an OD_600_ of 0.5, and allowed to grow for another three hours. Cells were then washed three times (5 min at 3.000 g, room temperature) with phosphate buffered saline (PBS) and transferred to minimal medium without nitrogen at an OD_600_ of 0.5. Cultures were incubated at 30°C, 200 rpm, and samples were taken at the indicated time-points by centrifugation. Samples were immediately used for RNA extraction or frozen in liquid N_2_ and stored at −80°C until use. For feeding experiments, nitrogen sources were added after these four hours of starvation, and RNA was then isolated from these cultures as described.

For RNA isolation, the RNeasy kit (Qiagen) was used according to the manufacturer's instructions. RNA quality was determined using a BioAnalyzer instrument (Agilent), and samples were labeled using the Quick Amp Labeling Kit (Agilent) with Cy5-CTP (GE Healthcare). As a common reference, Cy3-CTP (GE Healthcare) labeled RNA of an exponentially growing *C. albicans* liquid culture in YPD was used. Sample and common reference were co-hybridized on *C. albicans* oligonucleotide microarrays (ClinEuroDiag) and scanned using a GenePix 4000B scanner (Molecular Devices) and the GenePix software, version 4.1 (Molecular Devices). The data was normalized and evaluated using the GeneSpring GX software package, version 12.1 (Agilent). GO-Term analysis was performed with the same program, based on the genome and annotations from the *Candida* Genome Database [27]. All transcription data was deposited at the ArrayExpress database (www.ebi.ac.uk/arrayexpress) under the accession number E-MTAB-2171.

### Microarray data analysis and network inference

Analysis of raw microarray data was performed using the “Limma” package [28] of the statistical language “R” [29]. Spatial effects in each array were corrected by “LOWESS” normalization, and between-array normalization was performed using the “Quantile” method. Differentially expressed genes were identified using Empirical Bayes statistics [28] with a false-discovery rate of 0.01. Hierarchical clustering by the agglomerative average linkage method was performed in order to identify time points with similar expression profiles.

As the number of possible network structures grows exponentially with the number of genes in the network, we cannot reliably infer interactions between a large number of genes. For this reason, a number of genes was selected to be included in the models based on both, expression data from this work and literature knowledge. Genes which were found significantly up- or down-regulated were selected if they had a possible role in nitrogen metabolism, based on known *C. albicans* gene functions and *S. cerevisiae* homologs.

Prior knowledge comprises possible interactions from literature and other data sources. Several studies have shown that prior knowledge improves the reliability of the network inference approach [20]. A confidence score was attributed to each interaction based on the source of the data (high for repeatedly confirmed data from the literature, low for data inferred from suspected homologs in other species). Prior knowledge was softly integrated into the networks, allowing new, strongly supported interactions to overrule prior knowledge data [22]–[24]. Table S1 shows the prior knowledge information used for network generation.

Given the selected genes to be included in our models, their measured expression data, and putative interactions in the prior knowledge, three regulatory interaction networks (N-starvation, arginine feeding, BSA feeding) were predicted using the Net*Gene*rator tool [30], [31]. Net*Gene*rator is based on differential equations and models the expression of a gene as the weighted sum of all the other genes and an external stimulus [30]. Non-zero weights define the network, where a positive weight is interpreted as an activation and a negative one as a repression. Net*Gene*rator uses a heuristic search strategy to optimize the weights for three criteria: a) the simulated model fits to the measured time series data b) the number of interactions (non-zero weights) is minimized, *i.e.* only the most important interactions to fit to the data are reported, and c) the predicted interactions (edges) fit, if possible (*i.e.* softly integrated) to the prior knowledge.

We augmented the standard Net*Gene*rator in two ways [22]–[24]. First, we checked whether the predicted interactions were robust against noise in the expression data. To this end, we repeated the inference approach 1000 times with added Gaussian noise (μ = 0, σ^2^ = 0.05). Second, we checked the dependence of the predicted interactions on prior knowledge by repeating the inference approach 1000 times with randomly skipping 5% of prior knowledge in each iteration. Only edges present in more than 50% of the predicted networks after both consistency checks were taken into account.

## Results and Discussion

**Figure 1:**
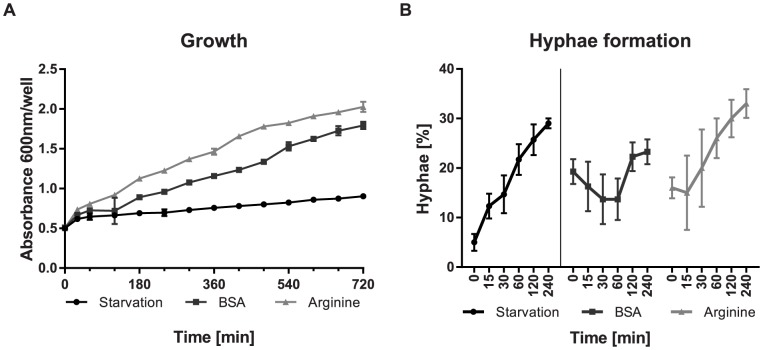
Growth and morphology of *C. albicans* under the experimental conditions. (A) Shortly after onset of nitrogen starvation, *C. albicans* growth rate is reduced to nearly zero. With nitrogen sources, growth resumes fast for arginine, and slower for BSA in the medium. (B) Formation of hyphae starts with onset of nitrogen starvation, and continues after addition of arginine for feeding. Under BSA supplementation, hyphae formation resumes after an early delay phase.

To determine the suitability of different nitrogen sources for fungal growth, *C. albicans* was grown individually with different nitrogen containing compounds. Media contained either one of the twenty proteinogenic amino acids, urea, or the complex compounds casamino acids (CAA) or bovine serum albumin (BSA). All nitrogen sources supported growth of *C. albicans* (not shown). Arginine was the amino acid to best support growth, similar to BSA or CAA as complex compounds. Hence, arginine and BSA were chosen for investigating amino acids and proteins as nitrogen sources, respectively. For BSA, growth was delayed initially, but the generation time shortened to levels comparable to growth in arginine at later time points (Fig. 1A). The BSA-fed cultures eventually reached a final optical density similar to the arginine-fed cultures. In medium without nitrogen, very little residual growth was observed.

Morphologically, the fungus switched to hyphal growth under nitrogen starvation and both, arginine and BSA feeding, as expected [32], [33]. Hyphae formation was more extensive during feeding with arginine, reaching up to 33% after four hours (Fig. 1B). This was also reflected by an increased transcription of hyphae-associated genes, as discussed below. Finally, supplementation with BSA led to a stalling of hyphae formation in the first hours, and a resumption at later time points (Fig. 1B). The presence of hyphae under all conditions allowed comparisons between the transcription profiles, and likely reflects the behavior of the fungus under *in vivo* conditions.

### Transcriptional profiling of nitrogen starvation and feeding

**Figure 2:**
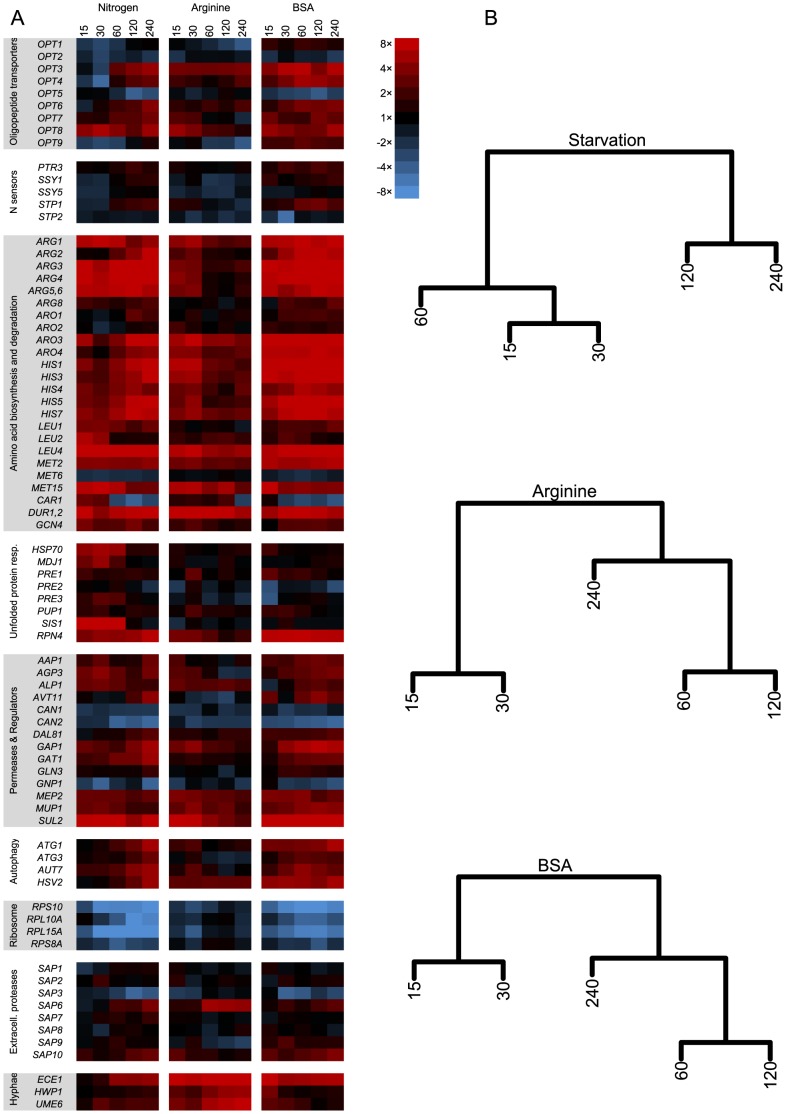
Heatmap of genes discussed in this work and clustering of time-points under different conditions. (A) Heatmap of relevant genes, arranged according to arbitrary functional categories. Expression levels are given in comparison to the onset of nitrogen starvation (0 min sample). For details, see the main text. (B) Hierarchical clustering of up-regulated genes at different time-points. A division can be seen between the early phase and the late phase, with the 60 min condition appearing in either, depending on the experiment.

Based on these growth experiments, microarray analyses were performed on *C. albicans* cells either starved for nitrogen, or grown in arginine or BSA after a period of starvation (Fig. 2A). Previous to starvation, cells were grown in YPD complex medium, to simulate an environment of relative nitrogen abundance from different sources similar to the gut. Overall, five time points were sampled in biological triplicates, in each of these experiments.In the following text, expression levels are always given in comparison to the onset of nitrogen starvation (0 min sample). Based on the expression profiles, the time-course samples can be divided into two major phases: an early ‘response phase’ (15 and 30 min) and a late ‘adaptation phase’ (120 and 240 min). The 60 min sample varies between these two, but most often clusters with the later time points (Fig. 2B), and therefore was added to the adaptation phase.

### Early response to nitrogen starvation

**Figure 3:**
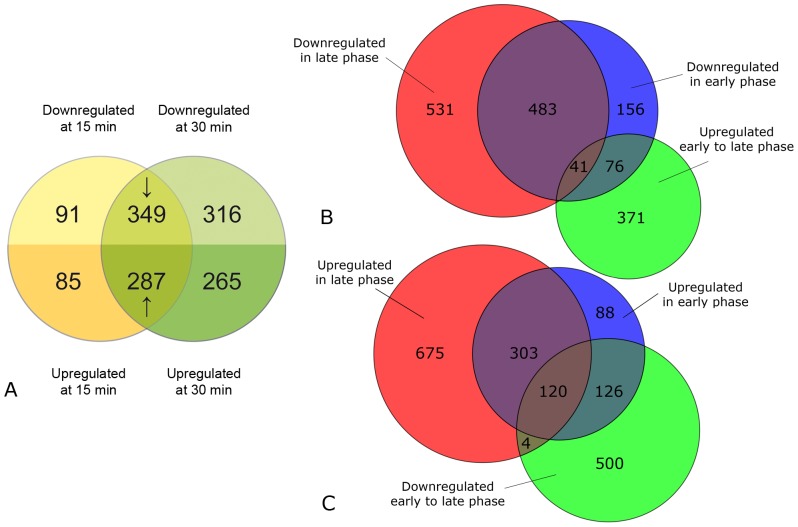
Overall transcriptional response to nitrogen starvation in response and adaptation phases. (A) 756 genes were significantly down-regulated (upper half of circles, ↓) and 637 up-regulated (lower half, ↑) at least two-fold during the nitrogen starvation response phase (030 min). Few genes were specific for the very early response (yellow, 15 min), and most were either common to 15 and 30 min of starvation or specific for 30 min (green). (B) Early down-regulation continues into the late nitrogen starvation response. Of the 756 genes which were down-regulated in the response phase (blue), a significant proportion stayed down-regulated as part of the late phase response (red). A smaller subset (117 genes) was among the genes which were up-regulated between early and late phase (green). Of those 41 were up-regulated between early and late phase, but still remained on a very low expression level compared to the onset of starvation. Additional 531 genes were specifically down-regulated in the late phase of starvation (red). (C) Early up-regulation is partly reversed in the later starvation phases, Of the 637 genes up-regulated in early phase of starvation (blue), about half stayed highly expressed in the late phase (red). In contrast, 246 genes were initially up-regulated, but their expression level was down-regulated between early (response) phase and the late (adaptation) phase (green). See text for more details.

Overall, under nitrogen starvation, 1393 genes were found to be differentially expressed during the response phase, either at 15 or 30 min, or both. Of the genes significantly and at least two-fold differentially expressed at 15 min (812), most (636) were still differentially expressed at 30 min, and, altogether, can be considered the core early response (Fig. 3A). This gene set comprises 287 up-regulated and 349 down-regulated genes.

### Immediate repression of protein biosynthesis and up-regulation of nitrogen metabolism

GO-term analyses of the down-regulated genes revealed a strong enrichment of genes involved in ribosome biogenesis (p<0.001), which on average were four-fold down-regulated at 15 min, and nearly six-fold at 30 min. At the same time, a strong unfolded protein binding response (GO:0051082) was observed in the up-regulated genes, possibly indicating a stall in translation as ammonium for amino acid biosynthesis became limiting. This includes genes encoding heat-shock proteins, like *HSP70*, and co-chaperones, like *MDJ1* and *SIS1*, the transcription of which increased up to 30-fold in the first 30 min (Fig. 2A). In *S. cerevisiae*, Rpn4 is a transcriptional activator of proteasome biogenesis [34]. The gene for its *C. albicans* homolog, *RPN4*, was up-regulated very early (more than threefold at 15 min) and the RNA levels remained high throughout the starvation period. Proteasome biogenesis accordingly increased, as genes for the constituting subunits (GO:0000502) were up-regulated early, peaking at 30 min after onset of starvation. Typical subunit genes include *PRE3* and most other *PRE* genes, and, to a lesser extent, the *PUP1* subunit gene. These transcriptional responses demonstrate an immediate sensing of the stress condition, and indicate a fast re-modeling of cell physiology to counteract the nitrogen starvation.

Nitrogen metabolism, mainly in the form of amino acid biosynthesis (GO:0008652), was likewise up-regulated during the early stage (p<0.001). Examples for up-regulated pathways include biosynthesis of arginine (*ARG* genes in Fig. 2A), aromatic amino acids (*ARO*), histidine (*HIS*), leucine (*LEU*), and methionine (*MET*). Accordingly, the gene encoding their transcriptional activator Gcn4 was found to be up-regulated more than two-fold already at 15 min. In baker's yeast, the transcription of *GCN4* likewise increases under nitrogen starvation. Yet, due to inhibition of Gcn4 translation via small untranslated upstream ORFs, its effector genes are unaffected [35]. The transcription of *ARO4* (and *HIS7*) in *S. cerevisiae* is even lower under general nitrogen starvation than under starvation for specific amino acids [35]. In contrast, in *C. albicans* the expression of the amino acid biosynthesis genes follows the transcript levels of *GCN4* more closely. This hints at a possible lack of *S. cerevisiae*-like translational regulation of Gcn4 activity in *C. albicans*, although the necessary regulatory upstream ORFs in the leader sequence of the gene seem to be present [36].

Similar to expression studies with *S. cerevisiae* [35], the urea amidolyase gene *DUR1,2* was strongly up-regulated at the onset of starvation and remained at this high level for the duration of the experiment. The expression of *DUR1,2* is indicative of arginine degradation, as is the transient up-regulation of the *CAR1* arginase gene. The oligopeptide transporter Opt1 was transcriptionally repressed only early after transfer from YPD, as were Opt4 (Ifc4) and Opt3 (Ifc3) at 30 min. In later starvation, the expression of these genes increased again to level reaching or exceeding the pre-culture. This indicates previous active transport via these peptide transporters during growth in YPD complex medium, which was shut down only early after transfer to starvation conditions. Interestingly, *OPT8* was up-regulated already at early time-points during nitrogen starvation, hinting towards a specific role of this transporter under nitrogen starvation.

Finally, the general amino acid permease genes *GAP1* and *AAP1*, and the ammonium permease gene *MEP2* were up-regulated in the early response, presumably allowing uptake of any nitrogen source that might become available. This was paralleled by an early up-regulation of *GAT1*, which encodes their transcriptional activator [37], [38]. Genes for specific permeases, as the asparagine and glutamine permease Gnp1 and the basic amino acid permeases Can1 and Can2, were down-regulated, while permease genes involved in sulfur-containing amino acids, as *ALP1*, *MUP1*, and possibly *AGP3* [39], were counter-intuitively up-regulated after the switch from full medium to nitrogen starvation. Similarly, the gene for a putative sulfate transporter, *SUL2*, was immediately up-regulated. This likely reflects the import of sulfate from the medium, after sulfur-containing amino acids of the pre-culture became limiting. Thus, similar to the oligopeptide transporters, transporters of most specific amino acids are down-regulated under nitrogen starvation and replaced by more general types of transporters.

### Late adaptation response to nitrogen starvation

The adaptation phase, between 60 and 240 min, was generally characterized by continued repression of the translation machinery and genes involved in nitrogen uptake (Figs. 2A & 3B). Yet, of all genes up-regulated in the early response phase, many were also found to decrease in expression significantly in the adaptation phase (Fig. 3C). These include genes for proteins involved in the unfolded protein response, like heat shock proteins and chaperones. In contrast, genes for proteins involved in uptake of amino acids were increasingly transcribed. Genes for histones involved in chromatin assembly (GO:0031497), which were down-regulated in the response phase, were up-regulated again during the adaptation phase. After peaking at 30 min, the overall expression of genes coding for components of the proteasome was decreasing until 240 min. Instead, genes involved in autophagy, like *AUT7*, *ATG1*, and *ATG3*, which are homologs to part of the cytosol-to-vacuole targeting (CVT) pathway in *S. cerevisiae*, were up-regulated in the later phase. The same pattern was found for orf19.1793, a homolog of the yeast microautophagy gene, *HSV2*. This indicates a switch from the rapid proteasomal degradation to autophagal processes for recycling nitrogen compounds during later starvation.

Overall, the vast majority of genes up- or down-regulated during the adaptation phase had already been regulated in the response phase. Genes down-regulated only during the late phase were mainly involved in oxidative phosphorylation in the mitochondria, hinting at a general reduction in aerobic energy production.

### Relief from starvation is marked by up-regulation of genes involved in amino acid metabolism and nitrogen uptake

After the starvation period, the *C. albicans* cells were fed either arginine or BSA as the sole nitrogen source. This simulated the change from a nutrient poor niche, for example the phagosome, to a nutrient-enriched environment. While there is an ongoing debate on whether the phagosome is indeed a place of nitrogen starvation, data for *e.g*. *C. glabrata* indicates this is likely the case [40]. In contrast to *C. glabrata*, *C. albicans* does not rely on autophagy during interaction with macrophages [41], likely because escapes these immune cells by forming hyphae. For *C. albicans*, such changes in nitrogen availability may hence occur, for example, during the escape from phagocytes.

The transcriptional responses to this feeding could again be divided into a response and adaptation phase, where the overall transcriptional changes occurred faster under arginine feeding than with BSA. Of the 884 genes significantly up-regulated between 0 and 240 min of the starvation period, most (578) were subsequently down-regulated at least two-fold during the 240 min of arginine feeding. With BSA feeding, only 20 were down-regulated within the same time period. Of those, 13 were shared with the set of genes down-regulated in presence of arginine (S4A). Similarly, of 818 genes down-regulated at 240 min of starvation, 406 were up-regulated after 240 min in arginine medium, and 61 were up-regulated when BSA was present (Fig. S4B). The gross transcriptional changes during starvation were therefore mostly reversed after four hours in arginine, but not in BSA containing medium, where fewer changes were detected. This likely reflects the faster sensing, uptake and metabolism of arginine by the cells as compared to the complex nutrient source BSA, where cells continued to starve for nitrogen until the protein was sufficiently degraded by the action of proteases.

### Early phase of feeding with arginine and BSA

The immediate response to arginine feeding was characterized by the up-regulation of ribosome biogenesis genes from their low levels during starvation. This process was much slower during BSA feeding. The autophagic processes were mostly reduced to pre-starvation levels quickly in the presence of arginine, but not in BSA-supplemented medium. In contrast, the transcript levels of most genes involved in amino acid biosynthesis stayed high during the first 30 min of arginine feeding, and during the entire 240 min of feeding with BSA. Notable exceptions were the arginine biosynthesis (*ARG*) genes, which were immediately down-regulated in response to arginine feeding.

The oligopeptide transporter genes, which were up-regulated in the late nitrogen starvation period, were generally down-regulated in the response and adaptation phases of arginine feeding (with the exception of *OPT3* which remained at its high post-starvation expression level). However, transcript levels for these transporters stayed high or even increased during BSA feeding, reflecting the different nature of the respective nitrogen sources. Similarly, genes for the general amino acid permeases, *GAP1* and *AAP1*, were down-regulated after a short initial up-regulation in arginine, but were continuously upregulated in BSA. Thus, they followed the changes in their transcriptional regulator, Gat1. Overall, the transcription profiles indicate a fast resumption of protein biosynthesis under arginine feeding, with concomitant reduction in amino acid synthesis and uptake efforts. In contrast, with BSA feeding, several hallmarks of the nitrogen starvation response stayed largely unchanged during the first four hours, in agreement with the slow initial growth with BSA.

### Late phases of BSA and arginine feeding are marked by differences in amino acid permease expression

The gene for the secreted aspartic protease, *SAP6*, was up-regulated in the late phase under both feeding conditions. *SAP6* is a member of a large protease gene family in *C. albicans* [14], which expression is known to be hyphal associated [42]. Therefore, the high expression of *SAP6* after addition of arginine is likely indicative of ongoing hyphal formation. This is supported by the up-regulation of further hyphae-associated genes, like *UME6*, *ECE1*, or *HWP1*. Similarly, expression of *SAP6* was up-regulated especially in the later phase of feeding with BSA, where hyphae formation was increasing. Under these conditions, the proteolytic activity of hyphae-associated Sap proteases may be critical for BSA degradation and hence, nitrogen uptake. It should be noted, that no probes for *SAP4* and *SAP5* existed on the microarrays used. Therefore, it is possible that all three hyphae-associated protease genes, *SAP4-6*, were expressed in the feeding experiments [42].

As described earlier, expression of the ribosomal genes resumed to pre-starvation levels in the arginine-fed culture, but not during BSA feeding up to 240 min. In addition, a continuing up-regulation of oligopeptide transporter genes was observed with addition of BSA, but not arginine. Amino acid permease and transporter genes such as *GAP1*, *AAP1*, and *AGP3*, were down-regulated after a short peak in transcription levels in the arginine adaptation phase, but up-regulated during BSA feeding. Thus, during the starvation phase for amino acids, general amino acid transporter genes are transcriptionally active, but these are down-regulated when a specific amino acid like arginine is offered. With BSA in the medium, the ongoing proteolytic activity likely liberated sufficient amounts of a broad range of free amino acids by that time to induce the up-regulation of general permease genes.

### Regulatory networks of nitrogen starvation and feeding

**Figure 4:**
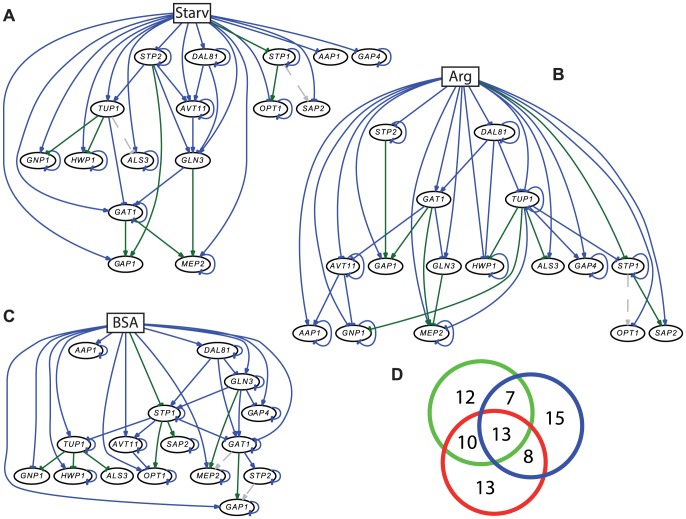
Predicted networks based on transcriptome data and prior knowledge under different conditions. (A) Nitrogen starvation network; (B) Arginine feeding network; (C) BSA feeding network. Blue lines indicate regulatory connections (edges) predicted by the transcriptional data alone, green lines show predicted connections in accordance with previous knowledge, and gray dotted lines indicate known interactions which were not found by the modeling algorithm with the current transcriptome data. (D) Overlaps in edges between the network. Green, nitrogen starvation; blue, arginine feeding; red, BSA feeding.

Our analysis of the expression profiles described above showed an adaptation to nutrient starvation, and a subsequent adaptation to different feeding conditions. Therefore, we used the same transcription data to create a regulatory network model of some of the most important interactions during nitrogen starvation. Similar networks, using the same genes, were created for arginine or BSA feeding. Representative regulator and effector genes were selected based on the transcriptome data and literature knowledge. The network dynamics were computationally modeled using the Net*Gene*rator tool (see Material and Methods). Fig. 4A shows the stable regulatory interactions during nitrogen starvation. The model-based kinetics fit very well to the measured expression values (Fig. S1). Overall, the network consists of 42 edges representing 26 gene-to-gene interactions and 16 influences of the environmental stimulus (nitrogen starvation) on gene expression. There are 11 activations and 15 repressions among the gene-to-gene interactions. Overall, this network is in agreement with previously known connections. Examples for this include the activation of *MEP2* transcription by Gln3 and Gat1 [38], or the increased transcription of *OPT1* by Stp1 [12]. Our network modeling did not detect the known repression of *ALS3* expression by Tup1, and the activation of *SAP2* by Stp1 (Fig. 4A). This is likely due to the fact that the expression of these genes increased only transiently under our experimental conditions. In contrast to *ALS3*, another hyphal marker gene, *HWP1*, plateaued at its expected high level after about 60 min, and its repression by Tup1 was correctly predicted.

Interestingly, the post-translationally modified transcription factors *Stp1* and *Stp2* [12] were found to be repressed in our network by the environmental stimulus, nitrogen starvation. Most likely, the transcription factors had been processed in the YPD preculture, which contains high levels of peptides and amino acids [12]. Under starvation, their proteolytic processing (and hence nuclear localization) stopped, and the yeasts required lower transcript levels of *STP1* and *STP2* to keep a steady cytoplasmic state of the proteins, explaining the down-regulation of transcription. We may have therefore detected posttranslational effects indirectly via transcript levels.

*DAL81*, on the other hand, was increasingly transcribed under the starvation conditions. The homolog in *S. cerevisiae* has been described as a pleiotropic transcription activator of nitrogen catabolic genes, and to amplify the transcriptional activation by Stp1 and Stp2 [43]. In our network, *DAL81*, together with *STP2*, activates orf19.7100 (*AVT11*). Avt11 is similar to a family of vacuolar transporters in *S. cerevisiae*, which act both in import and export of amino acids from and to the vacuole [44]. In *C. albicans*, *AVT11* has been described as possibly hyphae-associated [45]. This is not the case in our arginine feeding data, leaving the possibility of hyphae-independent function for this gene. Our network data indicates that Dal81 initiates (or aids Stp2 in the initiation of) nitrogen mobilization from the vacuole storage via Avt11. Alternatively, Avt11 may serve in storing surplus amino acids gained from starvation-induced proteolytic processes, as suggested for *S. cerevisiae* [44]. Other novel interactions, like the repression of *GLN3* expression by Dal81 warrant further investigations.

Fig. 4B presents the network after the nitrogen-starved cells were fed with arginine (kinetics in Fig. S2). There are 43 edges, 27 gene-to-gene interactions and 16 direct influences by the change from starvation medium. Among the gene-to-gene interactions, we predicted 7 activations and 20 repressions. The network is in overall agreement with the literature data with the exception of the *OPT1* activation by Stp1 [12], which could not be detected by our network model. As an example for down-regulation of the proteolytic activity, the repression of *SAP2* appeared in the network as expected: Since its activator gene *STP1* is down-regulated directly by the presence of amino acids as described previously [12], it cannot up-regulate *SAP2*. Finally, the role of Stp2 in the regulation is diminished as compared to its more hub-like function in the nitrogen starvation network, where its repression from YPD levels down-regulated several target genes (Fig. 4A).

Interestingly, in contrast to the nitrogen starvation network, the putative vacuolar amino acid permease gene *AVT11* is not up-regulated by Dal81 in this network, and both are repressed by the presence of arginine either directly or indirectly. This indicates that under arginine feeding, vacuolar nitrogen storage or mobilization is not important, and the regulatory connections were therefore not present in our network. Instead, Dal81 activates *GAT1* transcription, possibly in concert with a factor which is not part of this network, analogous to its interaction with Stp1 and Stp2 [43]. On the other hand, the observed formation of hyphae is indicated by the down-regulated *TUP1* gene, which codes for a repressor of *ALS3*, *STP1*, *HWP1* and *GNP1*. Unexpectedly, *GAP4*, normally expressed during hyphae formation [46], appears as positively regulated by the hyphae repressor Tup1, based on our transcription data. Activation of genes by Tup1 is rare, but has been reported before [47]. Overall, this network indicates a small contribution of Stp1 to transcription activities, and a change of roles especially for Dal81 compared to the starvation network.

Finally, during BSA feeding (Fig. 4C), the network inference resulted in 31 stable gene-to-gene interactions and 13 direct influences by the stimulus (kinetics in Fig. S3). Overall, there are 9 activations and 22 repressing interactions. The network input, BSA feeding, directly repressed *STP1* expression, which seems to contradict established network connections [12]. Yet, this repression reflects the measured initial drop of *STP1* expression level from its high value after nitrogen starvation (Fig. 4A). The activation seems to take place via or in combination with *DAL81*, which is itself up-regulated under BSA feeding. As expected, Stp1 activates *SAP2* and *OPT1* expression, indicative of protein degradation and oligopeptide up-take. In fact, Stp1 takes a central role in this network, as it is also connected to *AVT11*, *TUP1*, and *GAT1*. Similar to the nitrogen starvation network, *AVT11* is up-regulated at least indirectly via Dal81. This strengthens the notion of a possible amino acid import (instead of export) function of Avt11 of amino acids into the vacuole, in this case for the surplus amino acids gained from digesting the external protein. The precise directionality and substrates of Avt11 will be an interesting field for future studies on the nitrogen metabolism of *C. albicans*.

Finally, known important interactions which are missing from this network are the activation of *MEP2* transcription by Gat1, and the *GAP1* up-regulation by Stp2. The Gat1-*MEP2* connection is typically present under nitrogen starvation conditions [38], and is therefore not required during growth with BSA. On the other hand, direct regulation by Gat1 during BSA feeding [37] is likely sufficient to explain up-regulation of *GAP1* expression.

### Comparison of Networks

With our experiments, we deduced gene regulatory networks (GRN), which can be seen as condition-specific manifestations of a gene regulatory grid (GRG) encompassing all possible regulatory interactions [48]. We found that in *C. albicans* some of these grid connections were specifically realized in only one or two networks, while others were present in all possible GRNs. The networks therefore differed depending on the available nitrogen source. We compared the different GRNs to assess the transcriptional response to nitrogen supply on the network connection level. Fig. 4D depicts the shared and specific interactions among the three networks.

Shared among all three networks are 13 connections. These ‘core’ edges are mainly auto-repressions which indicates a (self-)limiting of expression over time. In addition, direct interactions from the change in nitrogen supply consistently activated genes like *MEP2*, *GAP1*, and *GLN3*. This often represents a transient up-regulation, likely due to a short-term internal nitrogen depletion that may occur after the transfer to a new medium, to which the fungus is not adapted. Hence, the short-term reaction may be considered a ‘sampling’ of the environment, which stabilizes under general nitrogen depletion and switches to a more specific response for the nitrogen sources arginine or BSA.

Of a total of 42 connections, 12 (28%) are exclusive for nitrogen starvation. This includes the previously discussed Dal81-*AVT11* activation, which may support nitrogen storage mobilization under this condition, and several activations of genes by Stp2. Further 10 and 7 connections are shared with BSA and arginine feeding, respectively.

Similarly, during BSA feeding, 13 out of 44 (29%) edges are specific. Many of those involve regulation by Stp1, and the activation of the oligopeptide transporter gene *OPT1*. When protein is available, these connections allow the uptake of processed oligopeptides. Interestingly, the activation of *SAP2* by Stp1 is part of five interactions shared with the arginine feeding condition. Also among those was the repressing effect of Tup1 on the hyphal marker gene *ALS3* that indicates the induction of hyphae.

Finally, 15 out of 43 (34%) connections are specific for the arginine feeding GRN. These include specific activation edges from the start of arginine feeding (input) towards the amino acid permease genes *GAP4* and *AAP1*, which reflect the initial up-regulation after the change from starvation to arginine containing medium. However, other permease activations are shared with nitrogen starvation (for example *GAP1* activation by Stp2) or with both, starvation and BSA feeding (Gat1-*GAP1*). This indicates a very generic function of this activation edge, possibly due to an initial expression of Gap1 under any condition of non-optimal nitrogen supply. This expression is later modified according to the prevailing nitrogen source, as discussed for its later down-regulation in arginine medium. In summary, this is in agreement with the function of a less specific general amino acid permease. Thus, under all investigated conditions, the regulation of permease genes mirrored the nutritional status, with the specific gene-gene interactions depending on the available nitrogen source.

Overall, the network models we predicted from the transcriptome data are specific for the presence and type of external nitrogen sources. About 1/4-1/3 of all detected connections are specific for any given condition, only about 1/3 are present under all experimental conditions, and another 1/3 was shared by two conditions. This shows that the response to nitrogen starvation - and presence of different nitrogen sources - takes place on both, the level of individual gene regulation and the global network level. Most importantly, a large subset of the total interactions in a GRN is specific for a given condition and defines the adaptation of the fungus to a specific nutritional status.

One possible mechanism for this kind of network changes is, apart from post-translational modifications, the presence of additional proteins or factors that facilitate or inhibit transcription factor functions. In this context, Dal81 is known to increase the promoter binding capacity of both, processed and unprocessed Stp1 and Stp2 proteins in *S. cerevisiae* [43]. On the other hand, inner nuclear membrane proteins of the Asi family reduce the ability of unprocessed Stp1 and Stp2 to activate transcription in yeast [49]. Thus, such mechanisms can enable regulatory connections under one condition, and prevent them under the other. This adds another layer of dynamic changes to the network connections.

### Conclusion

For *C. albicans* and other fungi, the ability to realize specific gene regulatory networks from a broader selection of networks has clear advantages. Instead of a stereotypical response, the transcriptional output to any given environmental input can thus be modulated for a fine-tuned reaction. This modulation can depend on changes to the network connections themselves, *e.g.* due to previous environmental conditions or due to factors which are not directly linked to the condition under investigation – for example carbon starvation. For pathogens, this may play an important role in their interaction with the host, as stress conditions can change rapidly, but often coincide. For example, nutrient deprivation (for glucose and likely nitrogen) and oxidative stress is encountered in the phagosome [50], and oxygen and glucose limitation can be found in sections of the gut [51], [52]. Thus, networks which can flexibly alter their internal connections enable an adapted rather than a uniform response to changing conditions. For the researcher, the dynamics of networks as observed under different conditions may pose an experimental problem. Networks should therefore always be viewed as dynamic entities, and the presence or absence of certain network connections can depend strongly on the overall state of the microbial system under observation.

For our modeling, we have chosen representative genes for the different functions required for adaptation to nitrogen availability. Generally, using more data (time-points as measured with arrays) will lead to more reliable predictions of interactions, but at the same time raises the experimental and computational costs of the study disproportionally. However, in our approach we try to ameliorate the limitations of a small data set by modelling a sparse network where many parameters are zero. Thus only the most important interactions to fit to the data are shown. Additionally, we make use of re-sampling of randomly disturbed gene expression data and only predict interactions which are stable against disturbance. Finally, we integrate prior knowledge to guide the modelling approach and skip incorrect network structures. Using these genes with our approach, we have tested different scenarios of nitrogen availability and found several novel and potentially biologically relevant, flexible connections between these genes in our networks. These are now open for further investigations, for example with the help of targeted deletion mutants based on the network inference [24].

## Supporting Information

Figure S1 **Measured, interpolated and simulated expression kinetics of the selected genes under nitrogen starvation.** Data fit for the initial nitrogen starvation model before testing for robustness. Measured expression levels (log2) of all genes in the networks (circles) and the data interpolation (dashed line) are shown in addition to the model-simulated data (solid lines).
(TIF)

Figure S2 **Measured, interpolated and simulated expression kinetics of the selected genes under arginine feeding.** Data fit for the initial arginine feeding model before testing for robustness. Measured expression levels (log2) of all genes in the networks (circles) and the data interpolation (dashed line) are shown in addition to the model-simulated data (solid lines).
(TIF)

Figure S3 **Measured, interpolated and simulated expression kinetics of the selected genes under BSA feeding.** Data fit for the initial BSA feeding model before testing for robustness. Measured expression levels (log2) of all genes in the networks (circles) and the data interpolation (dashed line) are shown in addition to the model-simulated data (solid lines).
(TIF)

Figure S4 **Reversal of the transcriptional response to starvation during subsequent feeding with arginine or BSA.** (A) The 884 genes up-regulated at least twofold after 240 min of nitrogen starvation were mostly (578) down-regulated again under arginine feeding, but generally not during four hours of BSA feeding (20 genes). Only 299 of the genes continued to be up-regulated compared to the onset of starvation even after arginine or BSA feeding. (B) About half (406) of the genes with at least two-fold down-regulation under nitrogen starvation were up-regulated again under arginine feeding. BSA feeding had less effect, and the 61 genes up-regulated with BSA feeding overlap mostly (53) with the arginine feeding up-regulation.
(TIF)

Table S1 **Previous knowledge used for the generation of the networks.**
(XLS)
