# Supplementary figures and images for: Regulatory Networks Controlling Nitrogen Sensing and Uptake in Candida albicans
Source: PLoS One. 2014 Mar 20;9(3):e92734. doi: 10.1371/journal.pone.0092734 (PMC3961412; doi:10.1371/journal.pone.0092734)

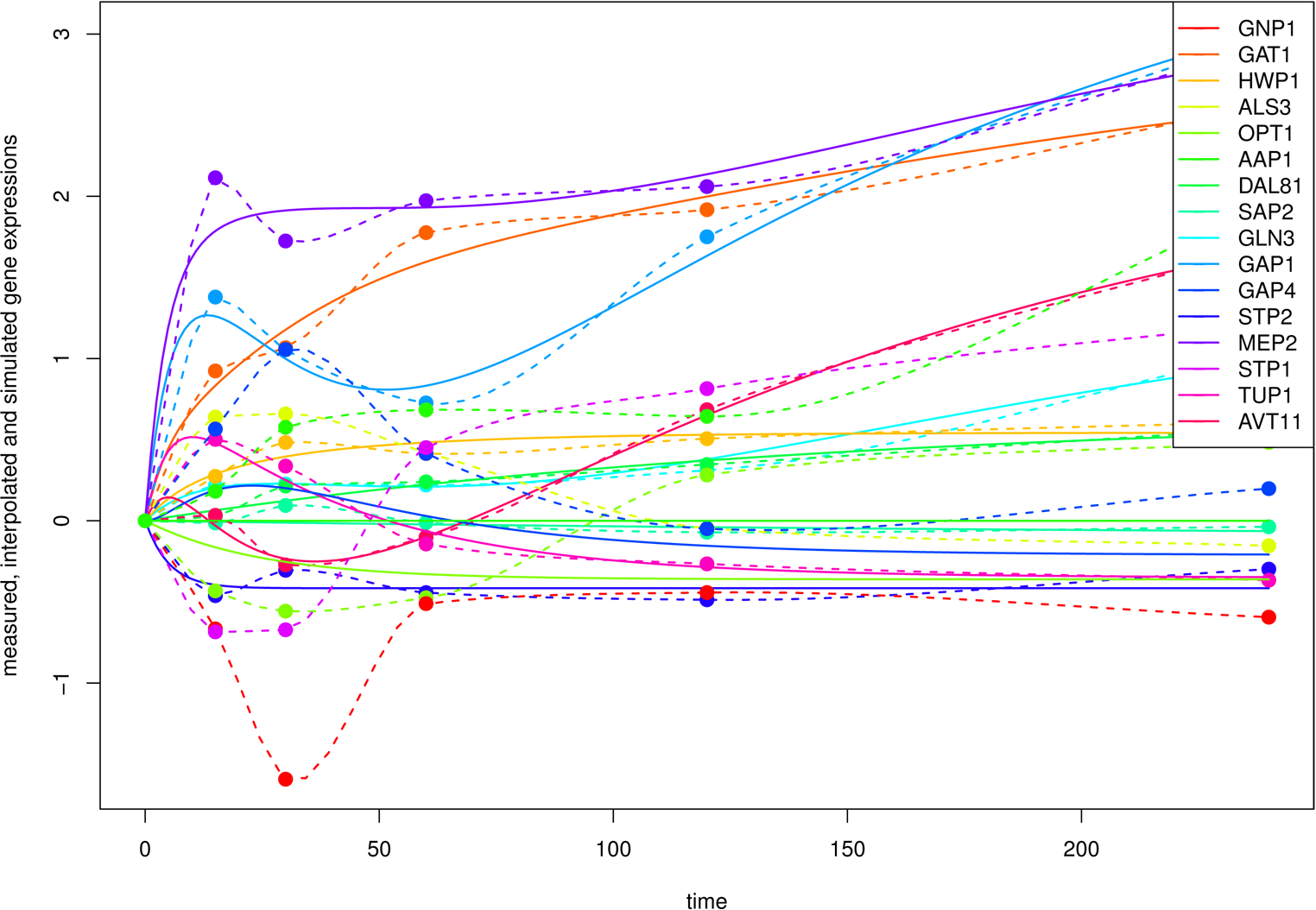

Supplement: Figure S1 — Measured, interpolated and simulated expression kinetics of the selected genes under nitrogen starvation. Data fit for the initial nitrogen starvation model before testing for robustness. Measured expression levels (log2) of all genes in the networks (circles) and the data interpolation (dashed line) are shown in addition to the model-simulated data (solid lines). (TIF) [file pone.0092734.s001.tif]

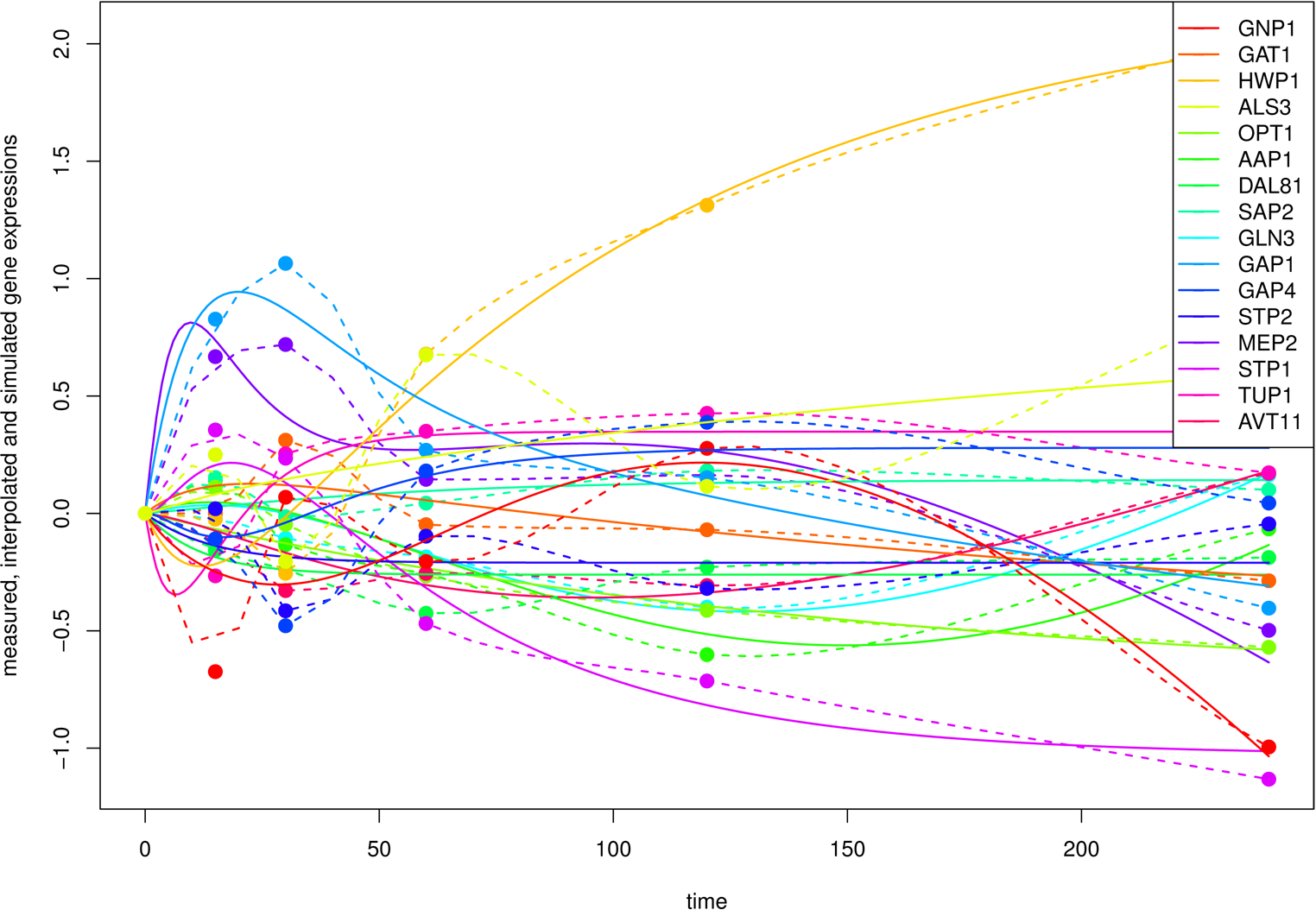

Supplement: Figure S2 — Measured, interpolated and simulated expression kinetics of the selected genes under arginine feeding. Data fit for the initial arginine feeding model before testing for robustness. Measured expression levels (log2) of all genes in the networks (circles) and the data interpolation (dashed line) are shown in addition to the model-simulated data (solid lines). (TIF) [file pone.0092734.s002.tif]

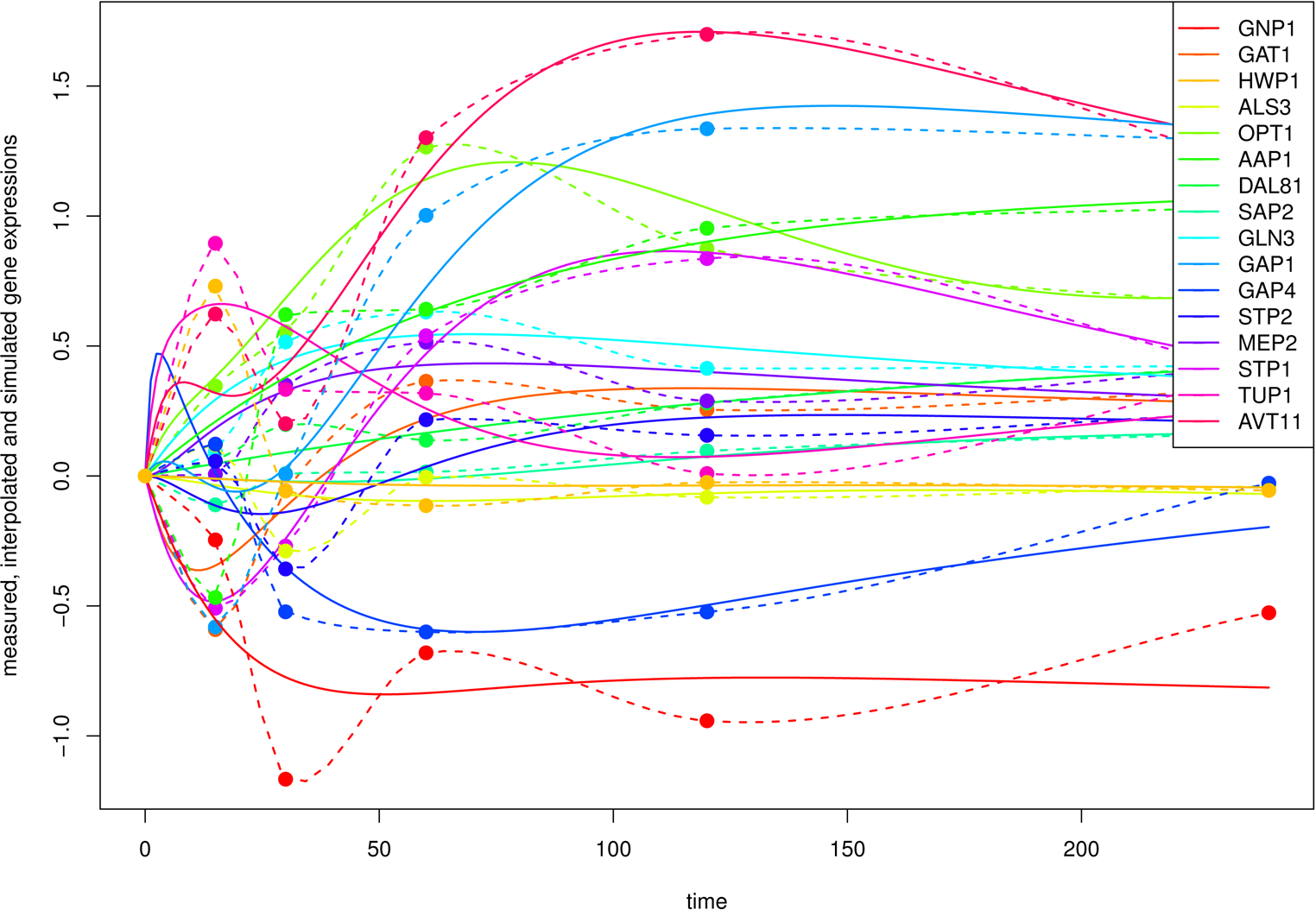

Supplement: Figure S3 — Measured, interpolated and simulated expression kinetics of the selected genes under BSA feeding. Data fit for the initial BSA feeding model before testing for robustness. Measured expression levels (log2) of all genes in the networks (circles) and the data interpolation (dashed line) are shown in addition to the model-simulated data (solid lines). (TIF) [file pone.0092734.s003.tif]

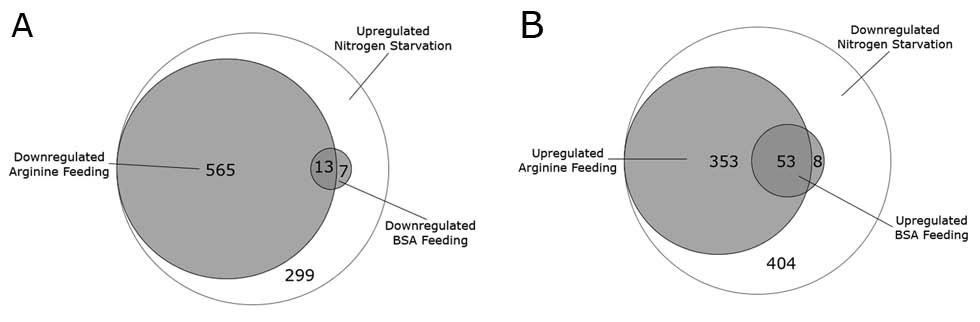

Supplement: Figure S4 — Reversal of the transcriptional response to starvation during subsequent feeding with arginine or BSA. (A) The 884 genes up-regulated at least twofold after 240 min of nitrogen starvation were mostly (578) down-regulated again under arginine feeding, but generally not during four hours of BSA feeding (20 genes). Only 299 of the genes continued to be up-regulated compared to the onset of starvation even after arginine or BSA feeding. (B) About half (406) of the genes with at least two-fold down-regulation under nitrogen starvation were up-regulated again under arginine feeding. BSA feeding had less effect, and the 61 genes up-regulated with BSA feeding overlap mostly (53) with the arginine feeding up-regulation. (TIF) [file pone.0092734.s004.tif]
